# Scrutinizing the Biomarkers for the Neglected Chagas Disease: How Remarkable!

**DOI:** 10.3389/fimmu.2016.00306

**Published:** 2016-08-11

**Authors:** Rosa T. Pinho, Mariana C. Waghabi, Fabíola Cardillo, José Mengel, Paulo R. Z. Antas

**Affiliations:** ^1^Laboratório de Imunologia Clínica, Instituto Oswaldo Cruz, FIOCRUZ, Rio de Janeiro, Brazil; ^2^Laboratório de Genômica Funcional e Bioinformática, Instituto Oswaldo Cruz, FIOCRUZ, Rio de Janeiro, Brazil; ^3^Centro de Pesquisa Gonçalo Muniz, FIOCRUZ, Salvador, Brazil; ^4^Faculdade de Medicina de Petropolis (FMP-FASE), Petrópolis, Brazil

**Keywords:** Chagas disease, biomarkers for immune responsiveness, human experimentation, clinical forms, mini review

## Abstract

Biomarkers or biosignature profiles have become accessible over time in population-based studies for Chagas disease. Thus, the identification of consistent and reliable indicators of the diagnosis and prognosis of patients with heart failure might facilitate the prioritization of therapeutic management to those with the highest chance of contracting this disease. The purpose of this paper is to review the recent state and the upcoming trends in biomarkers for human Chagas disease. As an emerging concept, we propose a classification of biomarkers based on plasmatic-, phenotype-, antigenic-, genetic-, and management-related candidates. The available data revisited here reveal the lessons learned thus far and the existing challenges that still lie ahead to enable biomarkers to be employed consistently in risk evaluation for this disease. There is a strong need for biomarker validation, particularly for biomarkers that are specific to the clinical forms of Chagas disease. The current failure to achieve the eradication of the transmission of this disease has produced determination to solve this validation issue. Finally, it would be strategic to develop a wide variety of biomarkers and to test them in both preclinical and clinical trials.

## Introduction

Population-based studies have identified a range of biomarkers that indicate exposure to, effects of, and genetic susceptibility for different pathogen-related diseases. These biomarkers could potentially be applied for diagnostic and prognostic purposes in human Chagas disease. The available data reveal the lessons learned to date and the current challenges that still remain to enable biomarkers to be employed reliably in risk evaluation for this disease. Our main purpose here is to revisit the current evidence and future trends in biomarker research for human Chagas disease. And due to recent, elegant systematic reviews focusing on this important topic (see below), we instead present here a literature review.

Chagas disease, or American trypanosomiasis, is caused by the etiological agent *Trypanosoma cruzi* and affects at least eight million people in Central and South America (1). The morbidity is high. The acute phase of infection is followed by an asymptomatic phase, but ~30% of infected patients develop a symptomatic, chronic phase that is characterized by either severe cardiac or digestive forms of Chagas disease (2, 3). Hence, the identification of consistent and reliable indicators of Chagas disease pathology, namely biomarkers or biosignature profiles, might facilitate the prioritization of management to those with a chance of contracting Chagas disease. Biomarker candidates might be engaged for the determination of some forms of Chagas disease. It is difficult to predict features related to morbidity and mortality as well as disease evolution. This information would assist in the supervision, decision-making, and follow-up related to this complex disease. Hence, there is a need to discover simple and quantifiable biomarkers that are more reliable than conventional screening methods and can be applied to support the diagnosis and prognosis of patients with heart failure (4).

A total of two systematic reviews of prospective biomarker targets during the course of therapeutic chronic Chagas disease underlined the requirement for the development of unique biomarkers to assess prompt responses to therapeutic management of the disease (5, 6). There are several studies that are investing in new diagnostic approaches to cure Chagas disease, particularly regarding the identification of disease biomarkers. Data from forthcoming studies will assist the categorization of patients in terms of clinical aspects for initial follow-ups (6).

Some blood-derived biomarkers that have demonstrated the capacity to predict the progression of early Chagas disease cardiomyopathy have been engaged to assess the value of anti-parasitic drugs and to identify initial cardiac and gastrointestinal injuries in asymptomatic patients. Nevertheless, future studies with extended follow-ups are required to establish biomarkers that are able to assess clinical or parasitological cures following therapy (6).

Biomarkers might be categorized based on biochemical configurations and major biological activities, such as inflammation and cellular injury biomarkers, metabolic biomarkers, prothrombotic biomarkers, and antigenic biomarkers (i.e., specific antigens of the parasite). Conversely, we propose a different classification. Table 1 and the next sections of this review summarize the recent data related to biomarker research. Once the scientific data become more readily available, the future identification of critical and consistent biomarker candidates for human Chagas disease should be simplified.

**Table 1:** **Summary of biomarker investigations related to Chagas disease**.

Study	Source	Biomarker name	Result	Reference
Experimental, parasitemia-specific	Antigenic	Aptamer	Increased levels	(39, 40)
Chagasic cardiomyopathy	Genetic	CCL2 and MAL/TIRAP	Increased susceptibility	(41)
Chagasic cardiomyopathy	Genetic	CCR5	Protection	(41)
Chagasic cardiomyopathy	Phenotype	CD15s+ Treg cells	Protection	(35, 36)
Chagasic cardiomyopathy	Phenotype	CD27+CD28+CD8+ T cells	Protection	(37, 38)
Non-specific	Plasmatic	TIMP-1 and TIMP-2	Increased levels	(5, 6)
Non-specific	Plasmatic	Troponin I	Increased levels	(5, 6)
Non-specific	Plasmatic	TGF-β	Increased levels	(5, 6)
Asymptomatic	Plasmatic	IL-10	Increased levels	(11)
Non-specific	Plasmatic	APOA1	Decreased levels	(7)
Non-specific	Plasmatic	Fibronectin	Increased levels	(7)
Asymptomatic	Plasmatic	MMP-2	Increased levels	(14)
Chagasic cardiomyopathy	Plasmatic	MMP-9	Increased levels	(14)
Chagasic cardiomyopathy	Plasmatic	ANP, BNP, N-terminal pro-BNP, IFN-γ, TNF-α, IL-1β, and IL-6	Increased levels	(4, 8, 10, 11)
Chagasic cardiomyopathy	Plasmatic	miRNA-1, miRNA-133a and -133b, and miRNA-208a and -208b	Decreased levels	(13)
Experimental, chagasic cardiomyopathy	Plasmatic	PICP and PIIINP	Increased levels	(15)
Experimental, chagasic cardiomyopathy	Plasmatic	Syndecan-4, ICAM-1, and Galectin-3	Increased levels	(16)
Efficacy	Management	KMP11, HSP70, PAR2, and Tgp63	Increased Ab. levels	(5, 6)
Efficacy	Management	Antigen 13 and SAPA	Increased Ab. levels	(5, 6)
Efficacy	Management	Tc24	Increased Ab. levels	(43)

## Plasmatic-Related Candidates

Effectively treated and cured chagasic patients may be identified based on their patterns of circulating biomarkers (7). Recently, studies have indicated that serum markers, such as A- and B-type natriuretic peptides (ANP and BNP, respectively), N-terminal pro-BNP, troponin I, TGF-β, MMP-2, and TIMP-1 and -2, are higher during the severe stages of Chagas disease and represent cardiac damage and inflammation. However, several candidates are not disease specific. Hitherto, the levels of these aforementioned natriuretic peptides have been found to be higher in Chagas disease patients with cardiomyopathy than in those with different forms or other etiologies. Moreover, natriuretic peptide levels are increased in asymptomatic chagasic patients who exhibit no signs of ventricular dysfunction. Hence, natriuretic peptides showed a high predictive value for evaluated outcomes (4, 8). Both of the pioneering studies were performed in Brazilian cohorts. According to the data from these studies, BNP is comparable to echocardiogram in terms of the assessment of cardiological patients. To reinforce those prior findings, another study performed in an independent setting found that BNP, pro-BNP, creatine kinase (CK)-MB, and MMP-2 have high predictive values for short-term mortality even in the presence of a decreased ejection fraction and other clinical signs of congestive heart failure, which were all found to be associated with severe chagasic cardiomyopathy in a Bolivian cohort (9). Because the BNP procedure is simple and quick, this biomarker can be used in endemic zones of Chagas disease with limited access to echocardiographic housing facilities. Finally, pro-BNP and *T. cruzi* DNA as detected by PCR are the only tests that have been found to have independent clinical value for disease staging in concert with electrocardiogram (ECG), echocardiogram, and clinical assessments (10). In a different study, the authors found that cytokine levels are related to cardiac injury in Chagas disease (11). Asymptomatic individuals exhibited high IL-10 levels that were associated with better prognosis. Conversely, IL-1β, IL-6, IFN-γ, and TNF-α reached the highest levels of expression in chagasic patients with cardiomyopathy. Overall, these findings sustain the perception that the balance between regulatory and inflammatory cytokines is associated with different forms of chronic Chagas disease (11, 12). Some micro (mi)RNAs, such as miRNA-1, miRNA-133, and miRNA-208, have been demonstrated to be involved with gene regulation properties and specific expression profiles, and imbalances can be found in chagasic cardiomyopathy (13). Recently, Santamaria and colleagues (7) pursued the identification of serum biomarkers that might be used as surrogates of therapeutic management in Chagas disease. APOA1 and specific fragments thereof and one fragment of fibronectin were uncovered. In chagasic samples, these biomarkers, excluding the full-length APOA1, are upregulated. These biomarkers revert to regular levels in 43% of cured patients. Notably, whenever serum MMP-9 levels are dominant, cardiac remodeling is strengthened and the advance of the cardiac form of Chagas disease is favored. Conversely, when serum MMP-2 levels prevail, patients persist as clinically asymptomatic. These processes might be IL-1β- and TNF-α dependent (14). In a particular model of infection, the cardiac levels of collagen I, III, and IV rise steadily and reach a peak during the chronic phase of Chagas disease. Thereafter, high serum levels of procollagen type I carboxy-terminal propeptide (PICP) and procollagen type III amino-terminal propeptide (PIIINP) are also observed. Given that increased PICP and PIIINP levels may indicate cardiac fibrosis, it is tempting to speculate that both biomarkers are suitable for detecting fibrosis during cardiac remodeling associated with *T. cruzi* infection (15). ICAM-1, galectin-3, and syndecan-4 have been found to be overexpressed in the hearts of mice chronically infected with *T. cruzi* (16). High levels of expression of galectin-3 in inflammatory cells have also been uncovered, and these levels are correlated with a decline in inflammation. A reduction in syndecan-4 and ICAM-1 might indirectly reduce cell migration into the myocardium and, thus, decrease inflammation (17). By contrast, in attempts to the uncover critical aspects of TGF-β as a candidate with prognostic value, several studies have demonstrated the influence of this anti-inflammatory cytokine on the development of chagasic cardiomyopathy by facilitating parasite cell invasion and its cycle (18, 19), improving parasite survival (20, 21), inducing exacerbated heart fibrosis and remodeling (22, 23), downregulating cardiac gap junctions (24), and mediating hypertrophy of the surviving cardiomyocytes (25). Increased circulating levels of TGF-β are observed in chronic Chagas disease patients (22, 26), and its active form is observed in the myocardia of chronic patients (27, 28). Moreover, due to the substantial involvement of TGF-β in the development of cardiac damage observed in Chagas disease, active compounds targeting TGF-β are currently under study as alternative treatments for the symptomatic cardiac form of Chagas disease (24, 29). Recently, the major cysteine protease from *T. cruzi*, cruzipain, has been observed to be capable of directly activating latent TGF-β, which favors parasite invasion into host cells (30). New therapeutic approaches for Chagas disease using anti-cruzipain compounds would be of beneficial not only due to their trypanocidal effect but also because they indirectly inhibit different TGF-β activities that are crucial for the development of Chagas disease. A retrospective study reported evidence supporting the clinical prognostic value of TGF-β as a biomarker for Chagas disease by demonstrating an association between its serological levels and clinical outcomes after 10 years of follow-up (31). Accordingly, TGF-β has demonstrated prognostic value as an independent predictor of all-cause mortality in patients without heart failure and with an ejection fraction above 45%. The optimal TGF-β cutoff for identifying patients who presented with all-cause mortality was 12.9 ng/ml. A further prospective study is clearly necessary to validate these data. Thus, the serological levels of TGF-β could be considered one potential biomarker for the outcome of Chagas disease and, moreover, could be used to follow the effects of treatments and interventions.

## Phenotype-Related Candidates

First, we would like to propose the regulatory T cell (Treg) axis as a biomarker for Chagas disease progression. As discussed elsewhere, a malfunction of regulatory immune mechanisms may also be involved in the pathogenesis of Chagas disease (32). This malfunction may be due to the action of Treg cells that have the potential to curb effector responses, allow a partially effective anti-parasite immune response, and therefore enable the establishment and maintenance of chronic Chagas infection (32). By contrast, recent findings in humans have demonstrated an increased rate of Treg cells in chagasic patients in the indeterminate chronic phase (free of disease) compared with those with heart damage, which suggests an important role for Treg cells in the control of the inflammatory response during Chagas disease (33). Additionally, using a non-depleting monoclonal antibody to CD25, it was recently demonstrated that Treg cells bearing the CD4+CD25+Foxp3+ phenotype may also help to control the inflammatory immune response in mice that are chronically infected with *T. cruzi* (34). Therefore, there is a clear indication that the functional activity of Treg cells might be of crucial importance during the chronic phase of the infection due to their potential to decrease tissue damage and pathology. Recently, the expression of CD15s (Sialyl Lewis x) was described to identify the majority of suppressive Treg cells in humans (35). Thus, this biomarker discriminates suppressive from effector CD4+CD25+Foxp3+ T cells in humans (35). Interestingly, a previous study reported that the expression of CD15s is decreased in peripheral blood lymphocytes from patients with severe Chagas disease (36). Although additional studies are urgently required to uncover the critical aspects of both phenotypes, the expression of CD15s in Treg cells may be a reliable biomarker for the prediction of the progression to pathology of chagasic patients. Additionally, another distinct phenotype, i.e., fully differentiated memory CD8 T cells (CD27−CD28−) bearing increased CCR7 expression, has been related to Chagas disease outcome (37). This study demonstrated an increase in total effector/memory CD8+ T cells in *T. cruzi*-infected individuals with mild heart disease compared with otherwise healthy controls. The study was based on the combined expression of CD27 and CD28 as previously proposed by Appay and colleagues (38), being related to a linear differentiation model for memory CD8+ T cells (38). This study suggested different fates of the T cell lineage, including early, intermediate, and late stages of cell-memory as follows: CD27+CD28+, CD27−CD28+ (or CD27+CD28−), and CD27−CD28− cells, respectively. As has previously been recognized, the proportion of fully differentiated memory (CD27−CD28−) in the total amount of CD8+ T cells is increased in mild Chagas disease. Conversely, the frequency of CD27+CD28+CD8+ T cells in the total memory CD8+ T cell population decreases as the disease becomes more severe. Albareda and colleagues (37) hypothesized that this pattern could be a consequence of the gradual clonal exhaustion of the CD8+ T cell population during infection. Analysis of the chemokine receptor CCR7 for lymph node homing (CCR7 expression) also revealed a significant increase in total effector/memory CD8 T cells in subjects with mild heart disease compared with healthy controls (37).

## Antigenic-Related Candidates

A correlated ELISA approach to the detection of aptamers in mouse plasma that is highly specific for circulating parasite excreted-secreted antigens (TESA) has been developed for biomarkers of *T. cruzi* infection (39, 40). An aptamer exhibited specific binding to TESA and trypomastigote extract, but it did not bind to self-proteins or *Leishmania donovani* proteins. Infected mice have exhibited increased levels of aptamer binding compared with control littermates, which suggests this aptamer as a potential candidate for a future biomarker of *T. cruzi* infection. Furthermore, this aptamer might sense circulating biomarkers in both acute and chronic phases of Chagas disease (39). Recently, the same group confirmed that *T. cruzi*-infected mice have considerably higher biomarker levels than their non-infected counterparts. This study found that the biomarker levels are also diminished upon therapy (40). However, the biomarker levels in the infected and treated group did not decrease entirely and persisted above the assay cutoff point, which suggests that parasitemia was reduced, but a cure was not achieved. The test was capable of distinguishing circulating biomarkers in animals infected with several subpopulations of *T. cruzi*.

## Genetic-Related Candidates

Genetic markers can provide evidence of the pathogenesis of Chagas disease and also have the potential to be utilized to identify new therapeutic targets. Frade and colleagues (41) studied genetic predispositions that influenced left ventricular ejection fractions in a Brazilian cohort. The authors found that CCL2 and MAL/TIRAP, but not CCR5, were linked to an increased susceptibility to chagasic cardiomyopathy.

## Management-Related Candidates

Regardless of most recent advances in drug development, there is virtually no consensus regarding the use of biomarkers to assess the efficacies of new drugs. Between the two main classes of recombinant proteins that are active during distinct ages and stages of Chagas disease, a 16-protein group and a combination of four recombinant proteins, namely KMP11, HSP70, PAR2, and Tgp63, have been identified (5). That combination could potentially serve as biomarkers candidates. Similarly, antibodies against antigen 13, among 5 others comprising the shed acute phase antigen (SAPA), have been demonstrated to be potential markers of cure efficacy [reviewed in Ref. (5)]. Additionally, a complement-mediated lysis test and an ELISA method based on Tc24 have also been created and the latter found to be a reliable candidate for a helpful parasite biomarker (42, 43).

## Future Trends

Researchers agree that the use of biomarkers in human Chagas disease will foster the progressing steps during the clinical assessment and also assist in the development of consistent diagnostic tools to lessen the time gap between the progression and detection of disease-relevant measures. Additionally, such biomarkers will allow for the prediction of both primary (genetic) and secondary (acquired factors) immunodeficiencies related to individual susceptibility. The deficiency of biomarkers in the prediction of parasitological outcome status and cure represents a main hurdle for the development of new drugs for Chagas disease. Thus, it is crucial to develop a reliable method to assess the cure of this disease. The aforementioned classes of biomarkers could yield uninterrupted longitudinal results related to Chagas disease management. Processes that are commonly used to identify biomarkers cannot be employed as endpoint evaluations in human clinical trials for ethical reasons. Some existing studies aim to develop alternative, emerging applications for biotechnologies based on the results of chagasic biomarker research. Furthermore, data from biomarker discovery research, such as the presence of TESA, could be used in vaccine development. In parasite-challenged vaccinated animals, TESA positivity could be an indication that the immune response was not appropriate to control the infection (39, 40). As a result of these struggles, the success of biomarker research in Chagas disease has not yet allowed for a better understanding of the disease risk from the clinical perspective. A range of issues exacerbates this frustration. One such issue is the incomplete validations of many biomarker candidates. Before conducting human trials, it is essential to identify and validate biomarkers that indicate cured patients. It is also necessary to evaluate the risks associated with the use of new agents in larger cohorts; thus, high-throughput biomarker procedures are needed. Ideally, approaching an accurate, rapid, and reliable point-of-care diagnostic tool in resource-limited, high-burden settings for Chagas disease through evaluation of biomarkers across that clinical spectrum in order to detect relevant pathogen-specific fingerprints will be of communal benefits, which undoubtedly outweigh the costs. The WHO predicted the eradication of Chagas disease transmission by the year 2010, but this goal has not yet been achieved. Indeed, the disease is spreading beyond locations in which it was originally endemic (1). It is crucial to understand the partial corroboration of simple biomarker technologies to avoid generating data that exceeds our ability to analyze the applications of novel technologies that are emerging from population studies. Finally, it would be strategic to develop a wide variety of biomarkers and to test them during preclinical and clinical trials.

## Author Contributions

RP and PA conceived and participated in the design and coordination of the manuscript. MW, FC, and JM provided helpful discussions and edited the manuscript. All authors wrote, read, and approved the final manuscript.

## Conflict of Interest Statement

The authors declare that the research was conducted in the absence of any commercial or financial relationships that could be construed as a potential conflict of interest. The reviewer CL and handling Editor declared their shared affiliation, and the handling Editor states that the process nevertheless met the standards of a fair and objective review.
